# The Influence of Regulation on Trust and Risk Preference in Sharing Communities

**DOI:** 10.3389/fpsyg.2020.01369

**Published:** 2020-06-30

**Authors:** Sarah Marth, Thomas Sabitzer, Eva Hofmann, Barbara Hartl, Elfriede Penz

**Affiliations:** ^1^Institute Marketing and Sales, University of Applied Sciences Wiener Neustadt, Wiener Neustadt, Austria; ^2^Competence Center for Empirical Research Methods, Vienna University of Economics and Business, Vienna, Austria; ^3^Institute for International Marketing Management, Department of Marketing, Vienna University of Economics and Business, Vienna, Austria; ^4^Department for Management and Economics, Faculty of Business and Globalization, Danube University Krems, Krems an der Donau, Austria; ^5^Institute for Marketing and Consumer Research, Vienna University of Economics and Business, Vienna, Austria

**Keywords:** sharing economy, communities, harsh regulation, soft regulation, trust, risk preference

## Abstract

Sharing within communities has gained popularity in recent years. However, taking part in a community also comes with a certain amount of risk. This perceived amount of risk can be contained by regulations within a community as well as by potential participants’ trust in the community and the other members. We argue for a relation between regulation and the willingness to take the risk of joining a sharing community with trust as a mediator. Thereby, we distinguish between two kinds of regulation (soft and harsh regulation) and two kinds of trust (implicit and reason-based trust) on two different levels (vertical and horizontal trust). In one laboratory and one online experiment with 432 participants overall, we found that the compound of high soft and low harsh regulation increases participants’ willingness to take the risk of participation and that the effect of soft regulation is mediated mainly by vertical and horizontal reason-based trust. Based on our results, we encourage sharing communities to count on soft regulation in order to increase potential members’ trust in the community and therefore take the risk to participate.

## Introduction

The sharing economy encompasses different social and economic practices and replaces ownership with the temporal access to goods (cf. Botsman and Rogers, 2010; Belk, 2014). Prominent examples of the sharing economy are online peer-to-peer (P2P) marketplaces, so-called *sharing-economy platforms* (e.g., Dillahunt and Malone, 2015), like Airbnb, an online marketplace facilitating accommodation sharing between private persons (Guttentag, 2015). Besides these P2P platforms, sharing activities are often organized in communities, which refer to one of the three centerpieces of the sharing economy (Acquier et al., 2017). In these communities, a good is shared, organized, and maintained by people who have in common that they all want to make use of the same goods (cf. Glover et al., 2005; Birky and Strom, 2013). This more social and less anonymous form of sharing can be referred to as sharing community (cf. Bardhi and Eckhardt, 2012) and comes with individual, societal, and environmental benefits (cf. Heinrichs, 2013; Kim et al., 2015). One example for such a sharing community is community gardens, in which a group of people share a piece of land, the tools, and often also the harvest (cf. Litt et al., 2011). Another example is housing communities sharing their own pool of cars, whereby all community members are responsible for the organization and maintenance of the shared cars (Hasan et al., 2018).

Sharing with strangers always implies risks, and especially sharing communities, which regulate themselves (Hofmann et al., 2017b), suffer from non-cooperative behavior. Some individuals try to take advantage of the shared goods without contributing anything and in this way exploit the community (Hardin, 1968; Hamari et al., 2016), for example, community members that leave a shared car dirty or with an empty fuel tank. Because associated risks and uncertainty are a barrier to participate in collaborative consumption (Lee et al., 2016), it is important to investigate how these risks can be minimized.

Research indicates that minimizing risks by using regulation and trust is essential to attract new members for participation (Slovic, 1987; Grabner-Kräuter and Kaluscha, 2003; Hamari et al., 2015). However, it is not completely clear how regulation, trust, and risks are linked to each other. The absence of regulation is related to several hazardous consequences, for example, high perceived risk (cf. Slovic, 1987), low participation (cf. Lowndes et al., 2006), conflicts (cf. Sabitzer et al., 2018), and distrust and low cooperation of community members (cf. Hartl et al., 2016). Regulation seems to be a key factor to prevent negative outcomes, which can be used as a guide to help members to behave properly in different situations. Further, some recent studies on sharing communities found that regulation is significantly related to trust (Sabitzer et al., 2018) and research on tax compliance showed that trust in the tax authority is related to regulation (Hofmann et al., 2017a). Therefore, we assume that regulation is a trust-building measure; first, it enhances trust, and in turn, people perceive participation as less risky, leading them to join the community. In this research, the effect of regulation and trust (as a possible mediator) on risk perception and actual risk-taking behavior is part of the analysis. The construct which is able to combine perceived risk and the decision to take this risk (joining the community) is called risk preference (cf. Charness et al., 2013). On the one hand, people prefer to be risk aversive, risk neutral, or risk seeking (Chiles and McMackin, 1996); on the other hand, external factors like the framing of a decision can change people’s risk preference (cf. Tversky and Kahneman, 1989; Frey et al., 2017). Thus, regulation could also change consumers’ risk preference, leading them to participate in the sharing community.

As past research on the sharing economy mainly investigated P2P platforms (e.g., Cannon and Summers, 2014; Edelman and Luca, 2014; Ert et al., 2016; Shaheen et al., 2017), this paper fills a gap by focusing on the under-researched domain of sharing communities, which according to Acquier et al. (2017) are one of the three foundational cores of the sharing economy. In addition, this paper contributes to theory and practice by extending knowledge about the relationship between regulation, trust, and risk preference. This becomes especially relevant in sharing communities in which no authority is present to shape people’s behavior in order to reduce risks for participation. Accordingly, the insights of this research can help to organize sharing communities, make potential members perceive participation as less risky, and hence facilitate their willingness to participate. Thus, we investigate if regulatory measures are able to change the risk preference of potential community members, leading them to join a sharing community. In a first study, the direct effect of regulation on risk preference is investigated using a laboratory setting with real consequences for participants. In a second study, the mentioned possible mediation effect of trust is examined.

In the next section, the theoretical background of sharing communities and the relations between risk preference, regulation, and trust will be discussed. Afterward, two studies are presented. Finally, we will discuss the paper’s results, limitations, and implications.

## Theoretical Background

### The Role of Sharing Communities

In the last decade, the term *sharing economy* has been used for several different organizational forms, which have in common that ownership is replaced by the temporary access to goods (Bardhi and Eckhardt, 2012). The sharing economy is defined as “consumers granting each other temporary access to underutilized physical assets (‘idle capacity’), possibly for money” (Frenken and Schor, 2017, pp. 4–5). To contribute to a clear definition of the concept, Acquier et al. (2017) proposed a framework, in which the sharing economy is used as an umbrella term for three organizational core parts, namely, the access economy, the platform economy, and the community-based economy. In this sense, sharing communities represent the community-based economy. Based on previous statements, we define a sharing community as a group of people whose members grant each other access to underutilized physical assets (idle capacity), whereby all community members are responsible for organization and maintenance of the shared goods.

In the sharing economy, previous research mainly investigated the access and platform economy (e.g., Cohen and Kietzmann, 2014; Zervas et al., 2017). Inexplicably, sharing communities are still the least investigated organizational form of the sharing economy. One possible explanation for this gap in research is that sharing communities still do not reach a critical mass to compete with traditional market alternatives (Bradley and Pargman, 2017). Although participation is still on a low level, the popularity of sharing communities rapidly increased over the last decade. For example, in Vienna, in 2010, only 13 community gardens were actively supervised by Gartenpolylog (2017a), an organization to support establishment of community gardens, while in 2019, the number already sextupled to 83 community gardens. Community-based sharing can have valuable outcomes for consumers, society, and the environment (Heinrichs, 2013; Puschmann and Alt, 2016). Participation facilitates equality and integration of disadvantaged people through social interaction and inclusion in a group (Wakefield et al., 2007; Litt et al., 2011). Besides social aspects, economic and ecological aspects also play a role. When people share and provide what they have, they can save money as they do not have to purchase the good they only need temporarily (Möhlmann, 2015). Furthermore, as a consequence, sharing grants the possibility for sustainable development as less goods have to be produced, and hence, waste is reduced (Puschmann and Alt, 2016). To conclude, sharing communities hold the potential to contribute to social, ecological, and economic sustainability (cf. Lozano, 2008) and support livability on a political, social and societal, individual, and educational levels (Gartenpolylog,2017b). Therefore, the proliferation of sharing communities should be fostered to make use of these potential positive outcomes of community-based sharing. However, uncertainty and perceived risks hinder people to take part in collaborative consumption (Lee et al., 2016), and without enough interested members, a sharing community cannot be formed. Thus, it is essential to investigate how the risk preference (perceived risks and risk-taking behavior) of consumers can be changed to attract new community members.

### Risk Preference

Risk is “a characteristic of decisions that is defined as the extent to which there is uncertainty about whether potentially significant and/or disappointing outcomes of decisions will be realized” (Sitkin and Pablo, 1992, p. 10). How much risk a person is willing to take determines their risk preference (Charness et al., 2013). People’s risk preference can vary between risk aversive, risk neutral, or risk seeking (Chiles and McMackin, 1996). Risk preference consists of both a stable trait component that explains about 50% of the variance and other factors (e.g., domain-specific or measurement-specific components) that explain the remaining variance (Frey et al., 2017). This entails that, at least partly, risk preference is influenced by variable factors, for example, through the framing of decisions that influence their domain-specific components (cf. Tversky and Kahneman, 1989). We expect that by varying information about used regulation in a sharing community, it is possible to shape people’s perception of risk and in turn their risk-taking behavior. Risk perception is defined as the subjective expectation of loss for choosing a particular option, which is important when making decisions under uncertainty (cf. Forsythe and Shi, 2003), and risk taking is a characteristic of consumer behavior and part of every decision (Bauer, 2001). In this paper, risk preference is not understood as eliciting a personal trait between risk aversion and risk seeking (cf. Chiles and McMackin, 1996) but as perceived risk and actual risk-taking behavior for two confronted options. Even though humans do not always decide rationally, in economic games, people usually decide for the option with the higher expected value or outcome (Simon, 1955). With the method of elicitation, the expected value for both options stays the same between experimental groups.

In recent literature, there is evidence that reducing the level of perceived risk is key to attracting new consumers for participation (Sheau-Fen et al., 2012). When people are confronted on whether or not to participate in a sharing community, they do not know if they will get the anticipated outcome. For example, gardeners do not know if other members will water their plants when they are on vacation. Thus, it is uncertain if the gardeners will reap what they sowed. People use subjective judgments to estimate the probability of an uncertain negative or positive result (cf. Kahneman et al., 1982). If potential community members estimate a high probability for a negative outcome and thus perceive high risk, it is unlikely that they will join the community, particularly if other less risky alternatives are available. Furthermore, research shows that people who expect a decision to have a profitable outcome are more risk tolerant (Grable, 2000); thus, their expectation shapes their risk preference. However, when people do not know what to expect, they rely on additional relevant information to make subjective judgments and assess the risk for choosing a specific alternative (Aven and Zio, 2011). We assume that by informing potential consumers of regulation procedures within sharing communities, it is possible to shape people’s expectation for a positive outcome and, thus, their risk preference.

### Regulation

One possible way to provide people with relevant information and reduce the level of perceived risk is through regulation (Slovic, 1987). Hence, the introduction of an appropriate form of regulation could be a way to shape peoples’ risk preference. Particularly, sharing communities lack in regulation and thus have a high demand for it (Hofmann et al., 2017b) to reduce risks and prevent community members from exploitation (cf. Hartl et al., 2016). Two forms of regulation can be distinguished: harsh regulation and soft regulation (Raven et al., 1998). Harsh regulation is defined by the strict setting up of rules and performing controls and sanctions when rules are not followed (cf. Raven et al., 1998). Soft regulation on the other hand is a supportive way to influence people’s behavior, based on a legitimized authority that possesses knowledge, has access to relevant information, and acts in a way people can identify with (Gangl et al., 2015; Hofmann et al., 2017b).

Current research indicates that soft regulation positively influences contributions to shared goods in public goods games (Hofmann et al., 2019). Other literature on regulation in the sharing economy shows that the perception of regulation differs between organizational models. Harsh regulation is often perceived as being part of the access economy, while soft regulation is perceived as equally high in the access, platform, and community-based economies (Hofmann et al., 2017b). Research also indicates that regulation in sharing communities is important but also that strict regulation should only be applied in severe cases, for example, if a member intentionally harms the community, such as through stealing or damaging something (Sabitzer et al., 2018). Too much use of harsh regulation might give the impression that others are not sticking to the rules, thereby diminishing trust in other members (Mulder et al., 2006).

### Trust

Besides the introduction of regulation, the establishment of trust is also important to reduce perceived risks and hence attract new members for participation (Slovic, 1987; Grabner-Kräuter and Kaluscha, 2003; Hamari et al., 2015). Trust can be defined as “a willingness to be vulnerable to another party” and “the willingness to take a risk” (Schoorman et al., 2007, pp. 346f.). Hence, the amount of risk someone takes depends on the amount of trust (Viklund, 2003; Schoorman et al., 2007). In sharing communities in which perceived risk and uncertainty are high at the beginning, as strangers come together to share goods, trust becomes essential, especially trust in other community members (Resnick and Zeckhauser, 2002; Kalkbrenner and Roosen, 2016; Mittendorf, 2018).

We distinguish between implicit and reason-based trust as proposed by Castelfranchi and Falcone (2010). Implicit trust develops automatically and unwittingly (e.g., through triggers such as similar social identities with the trusted party), whereas reason-based trust is based on rational and conscious cognitive processes in which internal and external factors influence whether to trust or not (e.g., competence and behavior of the other party) (Gangl et al., 2015). In addition, different forms of trust can be identified based on the hierarchical position of the entity which is trusted in. Hofmann et al. (2017b) differentiate between horizontal and vertical trust in the sharing economy. Horizontal trust represents trust in other members of the same hierarchical group, while vertical trust is the trust in an entity in hierarchically higher groups. In the case of a sharing community, there is a smooth transition between horizontal and vertical trust as vertical trust refers to trust in the community as a whole. In this paper, we take all aspects of trust into account and distinguish between implicit and reason-based trust on a horizontal level and vertical level. We assume that by generating trust and hence the confidence that others will not exploit one’s own vulnerability (cf. Schoorman et al., 2007), people are more willing to take the risk to participate in a sharing community.

### The Connection Between Regulation, Risk, and Trust

Perceived risk can be reduced by providing people with relevant information about regulatory measures (Slovic, 1987). Furthermore, Schoorman et al.(2007, p. 346) argued that “when the risk in a situation is greater than the trust (and, thus, the willingness to take risk) a control system can bridge the difference by lowering the perceived risk to a level that can be managed by trust.” Recent studies point out that especially soft regulation is effective in shaping people’s behavior in the sharing economy (Hofmann et al., 2019). Moreover, studies on sharing communities show that regulation and trust are significantly correlated, whereby a positive relation is found between soft regulation and reason-based trust (Sabitzer et al., 2018). Furthermore, a relation between regulation and trust was recognized in research on tax compliance, whereby again soft regulation was effective in changing people’s reason-based trust in the tax authority (Hofmann et al., 2017a).

As mentioned above, risk preference is influenced not only by regulation but also by trust (cf. Grabner-Kräuter and Kaluscha, 2003). “Trust is not taking risk *per se*, but rather it is a willingness to take risk” (Mayer et al., 1995, p. 712), which implies that these two concepts are inextricably linked to each other. Hence, risk preference seems to be dependent on the amount of trust (Viklund, 2003; Schoorman et al., 2007). Additionally, evidence on the relation between trust and risk derives from research on online markets. Consumers perceive a lower level of risk when they trust the online vendor, which leads to a higher purchase intention and in turn to more purchases (Grabner-Kräuter and Kaluscha, 2003; Kim et al., 2008). Similar mechanisms are expected to occur in sharing communities whereby trust in the community is expected to reduce perceived risks and hence increase people’s willingness to join.

**FIGURE 1:**
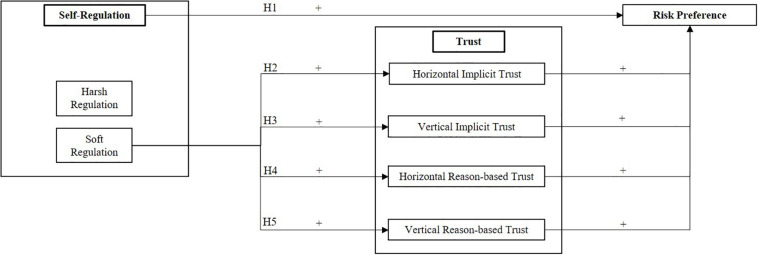
Research model.

Therefore, the question of whether regulation in a sharing community can be used to generate trust arises, which in turn affects people’s risk preference. In this sense, trust may influence the level of perceived risk and in turn the willingness to take the risk. From literature, it is not clear if risk preference is directly caused by regulation or mediated by trust. We propose the following hypotheses (also see Figure 1):

H1. Regulation increases the preference to take the risk of participating in the sharing community.

H2. Soft regulation increases horizontal implicit trust, which leads to a higher preference to take the risk of the sharing community.

H3. Soft regulation increases vertical implicit trust, which leads to a higher preference to take the risk of the sharing community.

H4. Soft regulation increases horizontal reason-based trust, which leads to a higher preference to take the risk of the sharing community.

H5. Soft regulation increases vertical reason-based trust, which leads to a higher preference to take the risk of the sharing community.

We start with the investigation of the direct effect of regulation on risk preference in a laboratory experiment, with real consequences for participants (H1). Afterward, the assumed mediation effect of trust will be investigated in an online experiment (H2–H5).

## Study 1

### Method

#### Sample

A convenience sample of 240 individuals was recruited via the subject pool of the Vienna University of Economics and Business. Six participants were excluded from the analysis because they did not follow the instructions and consequently their inputs could not be coded properly. Thus, the final sample consisted of 234 participants (60.3% women; *M*_age_ = 23.63, *SD*_age_ = 5.76). Most participants stated to have basic qualifications for university entry as their highest education (74.4%), and 73.1% reported to have a net income of less than 1000 euros per month. Most participants held a driving license (82.1%), and 47% stated to already have experience with car sharing. Furthermore, the participants stated to be rather environmentally conscious (environmental consciousness: *M* = 6.37, *SD* = 0.74), tend to consume environmentally friendly products (green consumerism: *M* = 5.06, *SD* = 1.25), stated to be moderately risk seeking (risk seeking: *M* = 3.96, *SD* = 1.41), and stated to be moderately trustful in general (trustfulness: *M* = 4.22, *SD* = 1.44; all scales from 1 “I don’t agree at all” to 7 “I totally agree”).

#### Procedure and Material

**FIGURE 2:**
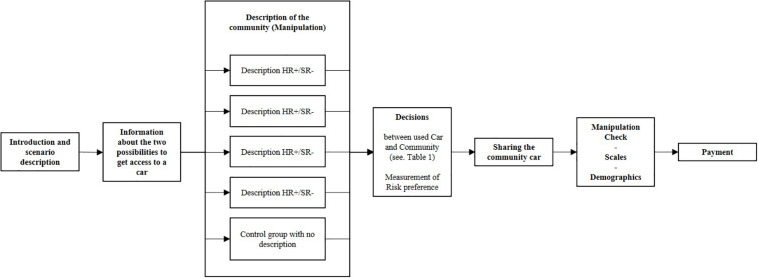
Flowchart of procedure with manipulation of high/low harsh regulation (HR±) and high/low soft regulation (SR±).

The procedure of study 1 is sketched in a flowchart in Figure 2. Below, the individual blocks of the diagram are described in more detail.

##### Introduction and scenario description

Participants were invited to the computer laboratory where 15 sessions with 8–20 participants were conducted. Participants took part in a laboratory experiment which was created with the software zTree (Fischbacher, 2007). In an introductory part, it was explained that all computers are linked to each other and that the participants would have to imagine sharing a car in a group of four people. They were told that these groups consisted of the participants in the room and that they would be randomly assigned to one of these groups. In addition, they were informed that financial remuneration depended on their own decisions, but also on the decisions of the other participants in the experiment. On the screen, participants received then the following information:

“Imagine, you run a delivery service and need a car for your work. Since you do not own a car but instantly need one, you consult two friends to evaluate the best options. You have 5000 experimental currency units (ECU) to invest in a car. Your friends tell you about two different possibilities.”

##### Information about the two possibilities

In the fictitious scenario, the 5000 ECU (1000 ECU were equivalent to a 1-euro payoff) could either be invested in a used car (with a probability of 10, 20, 30, 40, 50, 60, 70, 80, or 90% that the car would break down) or in a community car, which would be maintained and shared by the participant and three other community members (i.e., three other participants in the experiment) (see Table 1).

**TABLE 1:** Decision between used and community cars.

Probability that the used car breaks down shortly after purchase	I want to buy the used car	I want to use the community car
10%	?	?
20%	?	?
30%	?	?
40%	?	?
50%	?	?
60%	?	?
70%	?	?
80%	?	?
90%	?	?

It was explained that if the used car broke down shortly after the purchase, the investment would be gone but that if it did not break down, they earned 10,000 ECU with their delivery service and hence doubled their investment. Otherwise, if they decided for the community car, their earnings depended on the investment of all four community members, who shared the car. The overall investment of all four community members was also doubled and equally redistributed to all four participants. Participants earned 10,000 ECU in the case that all members invested their whole endowment of 5000 ECU.

The decision between the used car and the community car and therefore participants’ risk preference were measured using an adaptation of the multiple price list (MPL) method by Holt and Laury (2002), whereby a list with nine decisions between two alternatives is presented to the participants (see Table 1). In the MPL method, the possible outcome of both alternatives stays the same over all nine decisions; the only thing that changes is the probability of this outcome (Charness et al., 2013). Participants were informed that their payoff is dependent on their decisions in the MPL task. In this paper, we adapted the MPL method, whereby we changed the probability of the outcome of one alternative in all nine rows similar to the original task (for the used car), but the outcome and probability of the second alternative (community car) stayed unknown in all nine rows. This method allowed us to estimate perceived risk parameters of uncertain events as well as to measure actual risk-taking behavior.

Participants received the information that at the end of the study, the probability for the used car breaking down would be randomly determined by a blind draw of the participant out of a stack of nine different cards marked with the probabilities 10 up to 90%. If they drew, for example, a card with a 50% chance of the used car breaking down and they had opted for the used car at the 50%, a roll with a dice decided if the car actually had any damage. Otherwise, if they had opted for the community car, their payout depended on the investment of all four community members. Therefore, the design of the experiment allowed measuring the dependent variable *risk preference* (preference for either a known probability and possible outcome or an uncertain outcome, which is dependent on other people’s behavior).

##### Description of the community

Before asking participants’ to actually fill in the measure for risk preference (see Table 1), they received different information about the community. The community was described as using a combination of either high or low soft regulation (e.g., “The community is often/rarely available to members to discuss issues that arise”) and high or low harsh regulation (e.g., “The community has decided to monitor compliance with the usage rules often/seldom”). This resulted in an experimental two (harsh regulation: low vs. high) by two (soft regulation: low vs. high) between-subject design. Further, a control group without a description of the community was added. This group saw a blank screen for the time it took the participants of the other conditions to read the description. This finally resulted in five conditions.

##### Decisions between the used car and the community car

After reading the information about the community, participants were asked if they wanted to buy the used car or share the community car (see Table 1). This measurement of risk preference is an adaptation of the MPL method from Holt and Laury (2002). The combination with real monetary payoffs allows us to estimate perceived risk parameters of the uncertain event (outcome of the community car) and measure actual risk-taking behavior, all within the construct *risk preference*. In our adapted MPL experiment, participants (except extreme risk seekers) start with the used car and at some point, when they perceive the risk of the uncertain option as lower, switch to the community car (cf. Charness et al., 2013).

After making their decisions, all participants were instructed to imagine that they would be a member of the previously described community and would share a car with three other community members, or three other participants. This was necessary, in order to have all relevant information for the payment at the end of the study, even if the participant completely decided against the usage of the community car.

##### Sharing a community car

In the beginning of this part of the experiment, participants were introduced to the rules of sharing a community car, which was based on a public goods game (cf. Fischbacher et al., 2001). Participants were assigned to communities of four, and all four participants could decide how much of their endowment of 5000 ECU they wanted to invest in a shared community car. Afterward, the total investment of all four participants was doubled and equally redistributed to the participants. Three examples demonstrated these rules and how the result and final payout would be calculated. Subsequently, they decided how much they wanted to invest. On the next page, the overall investment of the four community members and the individual outcome were displayed.

##### Manipulation check, scales, and demographics

After the experimental part, participants filled in a questionnaire consisting of the scales harsh regulation (three items, e.g., “The community punishes strictly”) and soft regulation (seven items, e.g., “The community passes on information comprehensibly”) (both used for manipulation check), reason-based trust in the whole community (vertical, seven items, e.g., “I trust the community, because it performs its tasks well”), reason-based trust in the other users (horizontal, seven items, e.g., “I trust the other members, because they perform their tasks well”), implicit trust in the whole community (vertical, three items, e.g., “Most of the time, I trust the community without thinking about it”), and implicit trust in the other users (horizontal, three items, e.g., “Most of the time, I trust the other members without thinking about it”), based on Hofmann et al. (2014). Additionally, risk seeking (four items; Colquitt et al., 2006, e.g., “I enjoy being reckless”), trustfulness (four items; adapted from Cattell, 2001, e.g., “I trust in others”), environmental consciousness (six items; Alsmadi, 2007, e.g., “I respect all efforts to maintain and preserve the environment”), and green consumerism (five items; Alsmadi, 2007, e.g., “I usually buy environment-friendly products”) were assessed. The survey was finished with questions on the experience with the sharing economy and demographics. The internal consistency was satisfying for all scales (Cronbach’s α > 0.83).

##### Payment

After completion of the questionnaire, a chart was shown with the previously made decisions of the participant between the used car and the community car for the different probabilities of the used car breaking down (see Table 1), as well as their result of sharing the community car. Their payment was determined as described above, based on the drawn card, their corresponding decision, and the dice roll. The result of the used car was either 0 ECU/euro (if it broke down) or 10,000 ECU, or 10 euros (if it did not break down). The result with the community car was also 10,000 ECU, or 10 euros, if all other community members contributed the whole amount of their endowment. In the case where participants earned less than five euros in the experiment, they were still paid five euros as a show-up fee. However, they did not know about this show-up fee until the end of the study in order to make sure this does not influence the participants’ decisions in the experiment. The average payment of all participants was 7.80 euro.

### Results

First, in order to test if the manipulation of harsh and soft regulations worked as intended, a MANOVA in a two (condition harsh regulation: high vs. low) by two (condition soft regulation: high vs. low) design with the scales harsh regulation and soft regulation as dependent variables was calculated. The results showed that participants in the condition of high harsh regulation (HR+) ascribe significantly higher levels of harsh regulation to the car-sharing community than participants in the condition of low harsh regulation (HR−) [*M*_HR+_ = 4.95, *SD*_HR+_ = 0.17; *M*_HR__–_ = 2.27, *SD*_HR__–_ = 0.17; *F*(1,232) = 131.04, *p* < 0.01, η_*p*_^2^ = 0.36]. Also, participants in the condition of high soft regulation (SR+) rate the car-sharing community to operate with higher levels of soft regulation than participants of the low soft regulation condition (SR−) [*M*_SR+_ = 4.76, *SD*_SR+_ = 0.11; *M*_SR__–_ = 3.41, SD_SR__–_ = 0.11; *F*(1,232) = 78.10, *p* < 0.01, η_*p*_^2^ = 0.25]. These results indicated that the manipulation worked as intended.

**FIGURE 3:**
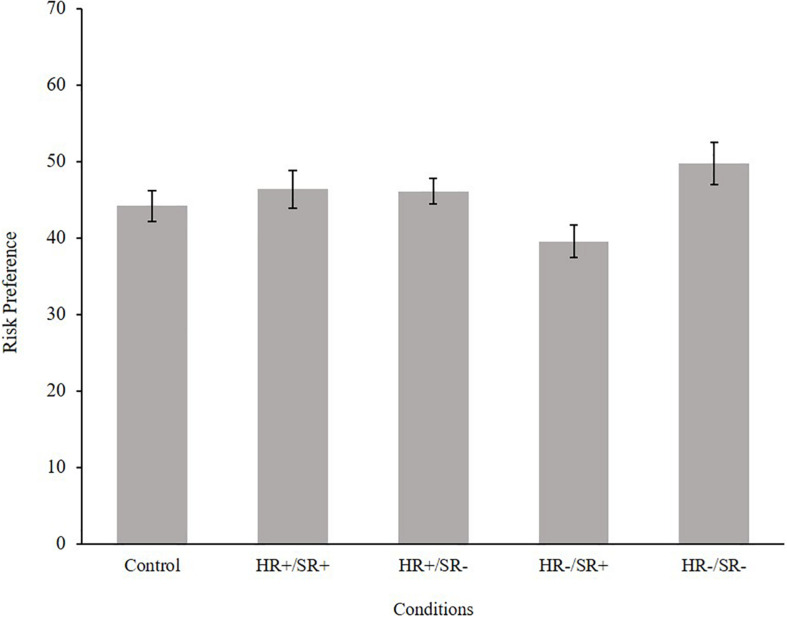
Means and standard error of risk preference per condition.

Afterward, to test the hypotheses that regulation increases the willingness to take the risk of the car-sharing community instead of the used car (H1), an ANOVA in a two (condition harsh regulation: high vs. low) by two (condition soft regulation: high vs. low) design was calculated. Thereby, participants’ answer on the MPL was chosen as a dependent variable (risk preference). The ANOVA showed that high soft regulation leads to a significant higher willingness to take the risk of the car sharing community instead of the used car on the MPL [*F*(1,189) = 4.85, *p* < 0.05, η_*p*_^2^ = 0.03]. The effect of harsh regulation was not significant [*F*(1,189) = 0.48, *p* = 0.49, η_*p*_^2^ < 0.01]. However, the interaction effect of soft and harsh regulation was significant [*F*(1,189) = 5.37, *p* < 0.05, η_*p*_^2^ = 0.03]. The means on the MPL of the actual five conditions (HR+/SR+, HR−/SR+, HR+/ SR−, HR−/ SR−, and control) revealed that participants in the condition of high soft and low harsh regulations prefer the uncertain risk of joining the car-sharing community at a lower probability of a breakdown of the used car than participants of the other conditions (*M* = 39.58, *SD* = 14.43) (see Figure 3).

Furthermore, a correlation analysis revealed a significant relation between soft regulation and vertical implicit trust (*r* = 0.18) and a highly significant relation between soft regulation and vertical reason-based trust (*r* = 0.28). In addition, soft regulation and risk preference were significantly related (*r* = −0.16), while harsh regulation showed no significant correlation with any kind of trust or risk preference (see Table 2).

**TABLE 2:** Means, standard deviations, and correlations.

	1	2	3	4	5	6
1 Harsh regulation						
2 Soft regulation	–0.02					
3 Implicit trust vertical	0.01	0.18*				
4 Reason-based trust vertical	–0.01	0.28**	0.61**			
5 Implicit trust horizontal	–0.02	0.05	0.60**	0.45**		
6 Reason-based trust horizontal	0.04	0.10	0.42**	0.70**	0.50**	
7 Risk preference	0.05	−0.16*	–0.13	–0.14	–0.08	0.03

*N* = 191. *p < 0.05, **p < 0.001.

### Discussion

Based on these results, we conclude that the combination of high soft regulation and low harsh regulation influences people’s risk preference the most and increases people’s willingness to take the risk of a sharing community instead of a used car. In experiment 1, due to the necessity of playing the public goods game in order to get a realistic scenario, the questionnaire about trust could only be implemented afterward. At this point, participants already knew the results of the public goods game, the contributions of the other players, and how much money they would earn when leaving the laboratory. We assume that this knowledge had a non-negligible influence on participants’ answers to the trust scale. However, the results of the ANOVA and correlation analysis indicate that soft regulation is an important factor on risk preference and participation in a sharing community and correlates with implicit and reason-based trust on the vertical level. Therefore, in order to specifically test if trust mediates the relationship between soft regulation and risk preference (H2–H5), a second study was conducted.

## Study 2

### Method

#### Sample

A convenience sample of 220 individuals was recruited via the subject pool of the Vienna University of Economics and Business. Twenty-two participants were excluded from the analysis because they stated to have taken part in study 1 and therefore have knowledge that could have influenced their answers in study 2. The final sample consisted of 198 participants (63.1% female; *M*_age_ = 24.45, *SD*_age_ = 6.00). Most participants stated to have basic qualifications for university entry as their highest education (63.6%), and 65.7% reported to have a net income of 1000 euros or less per month. About two-thirds (66.7%) reported living in a city with more than 300,000 inhabitants. The majority of participants held a driving license (86.9%), and 33.3% already used car sharing at least once. Furthermore, the participants stated to be moderately risk seeking (risk seeking: *M* = 3.38, *SD* = 1.30) and moderately trustful in general (trustfulness: *M* = 3.99, *SD* = 1.33).

#### Procedure and Material

The fictitious scenario was similar to that in study 1. Participants took part in an online experiment and were asked to imagine that they operated a delivery service but did not own a car. They were told that they had 5000 monetary units (ECU), which they could either invest in a used car (with a probability that the car breaks down) or in a community car, which was shared between the participant and three other people. They were informed that, if the used car broke down, the investment was gone. However, if it did not break down, they would earn 10,000 ECU with their delivery service (investment is doubled). Otherwise, if they decided for the community car, their earnings depended on the overall investment of all four community members. Before asking about their decision, they were told that they visited the website of the community and received information about the community, which was displayed in the discussion forum. The community was described as using either low soft regulation (e.g., “The community is *rarely* available to members to discuss issues that arise.”) or high soft regulation (e.g., “The community is *often* available to members to discuss issues that arise.”).

Participants were randomly assigned to one of the two experimental conditions, resulting in a between-subject design (low vs. high soft regulation). After reading the information about the community, they were asked if they would buy the used car or use the community car, for different probabilities that the used car could break down (see Table 1). After their decisions, participants filled in a questionnaire consisting of the same scales as in study 1, namely, reason-based trust in the whole community (vertical, seven items), reason-based trust in the other users (horizontal, seven items), implicit trust in the whole community (vertical, three items), and implicit trust in the other users (horizontal, three items), based on Hofmann et al. (2014). Additionally, the variables risk seeking (four items; Colquitt et al., 2006) and trustfulness (four items; Cattell, 2001) were assessed. The survey finished with questions on car-sharing experience and demographics. The internal consistency was satisfying for all scales (Cronbach’s α > 0.86).

### Results

**FIGURE 4:**
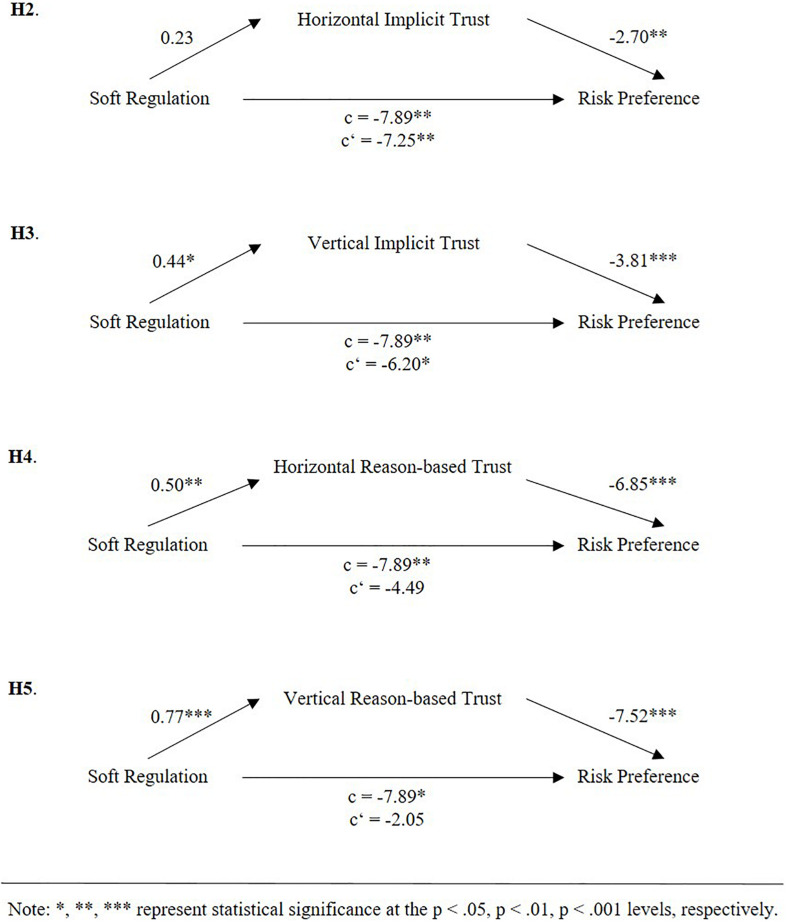
Results of mediation analyses.

In order to test Hypotheses 2–5, mediation analyses were performed with PROCESS (Hayes, 2017) (see Figure 4 for an overview). First, we tested if horizontal implicit trust mediates the effect of soft regulation on risk preference (H2). In step 1 of the mediation model, the regression of soft regulation on risk preference, ignoring the mediator horizontal implicit trust, was significant [*b* = −7.25, *t*(195) = −2.67, *p* < 0.01]. Step 2 showed that the regression of soft regulation on the mediator (horizontal implicit trust) was not significant [*b* = 0.23, *t*(196) = 1.21, *p* = 0.228]. Step 3 of the mediation process showed that the regression of horizontal implicit trust (mediator) on risk preference was significant [*b* = −2.70, *t*(195) = −2.72, *p* < 0.01]. Step 4 of the analyses revealed that controlling for the mediator, soft regulation was a significant predictor of risk preference [*b* = −7.89, *t*(196) = −2.87, *p* < 0.01]. Based on the insignificant effect of soft regulation on the mediator, we conclude that horizontal implicit trust does not mediate the relationship between soft regulation and risk preference, because there is no connection.

In the case of vertical implicit trust (H3), we found a partial mediation. Thereby, the direct effect of soft regulation on risk preference was significant [*b* = −6.20, *t*(195) = −2.29, *p* < 0.05], as well as the paths of soft regulation on the mediator and the mediator on risk preference, respectively [*b* = 0.44, *t*(196) = 2.42, *p* < 0.05; *b* = −3.81, *t*(195) = −3.67, *p* < 0.001]. Also, the total effect was significant [*b* = −7.89, *t*(196) = −2.87, *p* < 0.01].

When testing if horizontal reason-based trust mediates the relationship between soft regulation and risk preference (H4), we found an insignificant effect when ignoring the mediator [*b* = −4.49, *t*(195) = −1.78, *p* = 0.077]. The regression of soft regulation on the mediator (horizontal reason-based trust) was significant [*b* = 0.50, *t*(196) = 2.81, *p* < 0.01]. Also, the regression of horizontal reason-based trust (mediator) on risk preference was significant [*b* = −6.85, *t*(195) = −6.83, *p* < 0.001], as was the total effect [*b* = −7.89, *t*(196) = −2.87, *p* < 0.01].

Finally, we tested for vertical reason-based trust mediating the relationship between soft regulation and risk preference (H5) and found no significant direct effect between the two, when ignoring the mediator [*b* = −2.05, *t*(195) = −2.55, *p* = 0.422]. Again, the regression of soft regulation on the mediator (vertical reason-based trust) [*b* = 0.77, *t*(196) = 4.51, *p* < 0.001] and the regression of the mediator on risk preference [*b* = −7.52, *t*(195) = −7.46, *p* < 0.001] was significant. Therefore, based on this results and the significant total effect [*b* = −7.89, *t*(196) = −2.87, *p* < 0.05], we conclude that the relationship between soft regulation and risk preference is fully mediated by vertical reason-based trust.

When looking at means on the MPL of both conditions, we found that participants in the condition of high soft regulation (SR+) prefer the uncertain risk of joining the car-sharing community at a lower probability of a breakdown of the used car than participants confronted with low soft regulation (SR−) (M_SR+_ = 46.55, SD_SR+_ = 20.20; M_SR__–_ = 54.43, SD_SR__–_ = 17.87).

### Discussion

The second study allowed us to measure the proposed mediating influence of two kinds of regulation (soft and harsh regulations) and two kinds of trust (implicit and reason-based trust) on two different levels (vertical and horizontal trust). We tested the influence of soft regulation mediated by horizontal implicit trust, vertical implicit trust, horizontal reason-based trust, and vertical reason-based trust on the willingness to either take the uncertain risk of participating in a sharing community or buy a used car with a known risk of a breakdown (H2–H5). The results reveal that horizontal and vertical reason-based trust fully mediate the relationship between soft regulation and risk preference (H4 and H5). This means that the implementation of soft regulation mechanisms in a community leads to an increased trust in both the whole community (vertical) and its single members (horizontal), based on reasons like sharing the same values and goals. Furthermore, this increased reason-based trust leads to a higher willingness to take the risk of participating in the car-sharing community. In addition, vertical implicit trust has a partially mediating role (H3). So blindly trusting the whole community (vertical) partially accounts for the relationship between soft regulation mechanisms in the community and participants’ risk preference. However, blindly trusting (implicit trust) the other community members (horizontal) does not mediate this relationship; therefore, H2 could not be confirmed.

## General Discussion

Based on two studies, the current research shed light on the effect of regulation on risk preference and the mediating role of trust. Our results of study 1 show that regulation leads to a change of risk preference. As a result, the combination of high soft regulation and low harsh regulation, in particular, implemented in a sharing community increases people’s preference to take the uncertain risk of participating in such a community instead of taking the risk of purchasing a used car. This result confirmed Hypothesis 1. With the results of study 2, we were able to show that especially reason-based trust plays a significant role as a mediator in the relationship between soft regulation and risk preference. High soft regulation increases people’s reason-based trust in a sharing community, which leads people to a higher willingness to take the risk of participating in a sharing community (H4 and H5). This effect was not completely true for implicit trust. While horizontal implicit trust does not have a mediating role, vertical implicit trust allowed us to only partially explain the relationship (H2 and H3).

Recent studies showed that regulation and trust are significantly correlated (Hofmann et al., 2017a). However, so far, the direction of the relationship was unclear. The current paper is the first in the sharing economy to test the mediating influence of trust on the relationship between regulation and risk preference by using a controlled variation of soft and harsh regulations in experimental conditions and asking for different kinds of trust afterward. In consideration of our results, we feel confident to argue not only that the concepts of regulation and trust correlate with each other but also that the presence of certain regulations leads to an increase or decrease in trust. Our results indicate that the presence of soft regulation leads to increased levels of reason-based trust (horizontal and vertical) and increases implicit trust on a vertical level. Our results show that varying information about used regulation in a sharing community is able not only to influence people’s levels of trust but moreover to affect their perception of risk and then, again, their risk-taking behavior.

### Limitations and Future Directions

Despite the new insights of the current research, it also comprises some limitations.

First, besides regulation and trust, we did not include other factors which could influence people’s preference to take an uncertain risk in our analysis as control variables, especially the influence of individual factors, for example, gender, age, experience, and opinions on sharing economy offers as well as personality traits, like extraversion and openness, which are known influences on risk-taking behavior (e.g., Byrnes et al., 1999; Nicholson et al., 2005; Steinberg et al., 2008). Also, cultural differences in shared beliefs and values have been subject of research for decades and have been shown to influence people’s worldview and behavior. This is also true for people’s perception of risk and risk-taking behavior (e.g., Dake, 1991). We therefore assume that participants’ risk preference in a situation of decision similar to our studies’ scenario could vary depending on their cultural backgrounds. Considering our sample of mainly Austrian students, the generalizability of our results may be limited and should therefore only be transferred to other cultural settings with caution. Given the fact that sharing economy offers are available worldwide, we strongly encourage future research to deal with the relationship between regulation, trust, and risk preference in the sharing economy in different cultural settings.

Second, the convenient samples of both studies consisted mostly of students. Some authors argue that students behave differently than the general population, for example, in their behavior as consumers (Jones and Sonner, 2001). However, most consumers of offers and services in the sharing economy are between the ages of 18 and 29 and show a high educational level (PwC, 2015). The same is true for the participants in our samples and can therefore be presumed as a valid sample for studying the behavior of consumers of car-sharing offers. Considering the aging of most western populations, an increase of older consumers of car-sharing offers can be expected in the next years (Hartl et al., 2020). Therefore, future research may investigate the relationship between regulation, trust, and risk preference in the context of the sharing economy by employing a more diverse sample.

Third, the chosen experimental design has some limitations. Specifically, the experimental designs in the laboratory (study 1) and in an online setting (study 2), both using an artificial scenario, come with a limited ecological validity. Therefore, the generalizability of the results to a real-life scenario needs to be proven in further studies. Moreover, in both studies, the amount of regulation in the car sharing community was manipulated, while horizontal and vertical implicit trust and reason-based trust were assessed with a self-reporting scale after participants decided between the used car and the community car. On the one hand, this approach allowed testing the direct effect of regulation on risk preference. On the other hand, with this sequence of the experimental procedure, trust was only assessed after participants’ risk preference but was analyzed as a mediator of the relationship between regulation and risk preference. Future research on this topic should consider not only to manipulate harsh and soft regulation but also to manipulate the two kinds of trust (implicit and reason-based trust) on two different levels (vertical and horizontal trust).

Furthermore, our results show different effects of horizontal and vertical implicit trust. While horizontal implicit trust does not mediate the relationship between soft regulation and risk preference, vertical implicit trust partially does. As mentioned above, in communities, horizontal trust and vertical trust are special compared to other business models in which the trust in an authority (vertical) can be clearly distinguished from trust in other consumers (horizontal). However, there is a difference between trust in the whole community (vertical) and trust in other community members (horizontal), which can also be found in our results regarding implicit trust. It seems that high levels of soft regulation rather lead to implicit trust in other community members instead of trust in the whole community. We advise future research to consider looking into the effect of soft regulation on implicit trust in more detail as well as into the mediation of vertical and horizontal implicit trust on the relationship between soft regulation and risk preference as it is not clear why vertical implicit trust partially mediates the relationship, while horizontal trust does not at all.

### Conclusion and Implications

Our results suggest that soft regulation is one of the external factors which are considered in the cognitive processes when forming reason-based trust and can therefore be used as a trust-building measure and encourage people to participate in the community. Soft regulation reduces the level of perceived risk and direct people’s risk preference toward taking the risk of joining a sharing community instead of choosing alternative service and good providers. Therefore, we advise the members and initiators of sharing communities, like car sharing, book sharing, and food sharing communities and community gardens, who want to attract new members in the community to introduce and visibly use soft regulation and thereby foster trust among current members and potential new ones. Although sharing communities imply personal contact between members more often than other models in the sharing economy, like P2P sharing, the communication between members and the representation of a community to potential members are often handled online via a website. As such, the website of the community becomes relevant even before people decide to participate in transactions. Thereby, in order to reduce the level of perceived risk and enhance consumers’ attraction to a service, different trust-building measures can be implemented on websites (Bart et al., 2005). Our results suggest that information about high soft regulation within a community can be used as a trust-building measure, which can also be implemented on a website and therefore made visible to potential new members at an early stage of their decision to participate. However, the influence of regulation on trust may be important not only to attract new members but also to support cooperative behavior of existing members (Mayer et al., 1995). By trusting the other members of a community and being confident that others will not exploit one’s own vulnerability by acting uncooperative, again one can assume members to be more likely to act cooperative themselves. Besides visualizing soft regulation in a community on its website to attract new members, soft regulations, like information about knowledge and support of other members, can also be inserted in statutes, membership forms, and usage rules to be visible for active members and support a trusting and supportive sharing community.

## Data Availability Statement

The datasets generated for this study are available on request to the corresponding author.

## Ethics Statement

Ethical review and approval was not required for the study on human participants in accordance with the local legislation and institutional requirements. The patients/participants provided their written informed consent to participate in this study.

## Author Contributions

SM planned the studies, performed the analysis, collected the data, and co-wrote the manuscript. TS collected the data, performed the analysis, and co-wrote the manuscript. EH co-wrote and supervised the manuscript. BH planned the studies and co-wrote and supervised the manuscript. EP supervised the manuscript.

## Conflict of Interest

The authors declare that the research was conducted in the absence of any commercial or financial relationships that could be construed as a potential conflict of interest.
